# Incidence Rates of and Mortality after Hip Fracture among German Nursing Home Residents

**DOI:** 10.3390/ijerph15020289

**Published:** 2018-02-07

**Authors:** Hannes Jacobs, Hajo Zeeb, Falk Hoffmann

**Affiliations:** 1 Department of Health Services Research, Carl von Ossietzky University Oldenburg, 26129 Oldenburg, Germany; falk.hoffmann@uni-oldenburg.de; 2 Leibniz Institute for Prevention Research and Epidemiology—BIPS, 28359 Bremen, Germany; zeeb@leibniz-bips.de; 3 Health Sciences Bremen, University of Bremen, 28359 Bremen, Germany

**Keywords:** hip fracture, nursing home, incidence rate, mortality, health services research

## Abstract

Little is known about hip fracture rates and post-fracture mortality among nursing home residents. This retrospective cohort study examined incidence rates (IR) of and mortality after hip fracture in this population focusing on sex differences. A cohort of >127,000 residents ≥65 years, newly admitted to German nursing homes between 2010 and 2014 were used to calculate age-, sex-, care-need- and time after admission-specific IR. To determine mortality, the Kaplan-Meier-method was applied. Using Cox regression, we studied mortality and estimated time-dependent hazard ratios (HRs). For this purpose, to each person with a hip fracture, one resident without a hip fracture was matched by sex, age and care-need using risk-set sampling. 75% were women (mean age: 84.0 years). During 168,588 person-years (PY), 8537 residents with at least one hip fracture were observed. The IR for women and men were 52.9 and 42.5/1000 PY. For both sexes, IR increased with rising age and decreased with increasing care-level. IR were highest in the first months after admission and subsequently declined afterwards. The impact of hip fractures on mortality was time-dependent. Mortality of residents with hip fracture was highest in the first two months after fracture compared to those without (HR): 2.82; 95% CI 2.57–3.11) and after six months, no differences were found (HR: 1.10; 95% CI 0.98–1.22) Further research should always include analyses stratified by sex, age and time period after admission.

## 1. Introduction

The aging population in most industrialized countries, including Germany, and the high life expectancy associated with demographic changes are causing significant health and social care problems. In 2050, about 23 million people over the age of 65 will be living in Germany and the proportion of people being 80 years or older will have grown by 8% to 13% [1,2].

In this context hip fractures are a major public health problem that can result in an increased care need or mortality, especially for older people [3]. That is why nursing home residents are an extremely vulnerable population and have a high risk of experiencing a hip fracture compared to community-dwelling persons of the same sex and age [4,5,6]. Most previous studies show that women are more frequently affected by hip fractures than men [5,7,8]. However, there are also studies that show the same risk for women and men or even a greater risk for men, especially in nursing home residents [4,6,9]. Both in nursing home residents and community dwellers the incidence of hip fractures increases with rising age [10,11,12,13].

Another potential risk factor for developing a hip fracture is the functional status. Previous studies suggest a higher risk for people who have a higher degree of mobility. For example Walter et al. observed an increased risk in persons being able to transfer to and from bed, chair or toilet independently [14]. Berry et al. registered that rates of hip fracture decreased as activity of daily life (ADL) impairment increased [9]. In a German nursing home cohort, Rapp et al. observed an almost linear negative association between incidence of hip fracture and increasing care level [8]. However, in another study with a different study population, Rapp et al. showed similar fracture risks for residents with level of care ‘I’ and ‘II’ and a clearly lower risk in residents with level ‘III’ [15].

The time period between admission to a nursing home and the occurrence of a hip fracture seems also to be of interest. In Germany, two studies have shown that the risk is greatest in the first few months after admission to a nursing home [8,15]. However, this aspect is little discussed in the current literature and represents an important research gap.

Mortality rates in nursing homes are considerable and male sex is significantly associated with death in patients with hip fracture [9,16,17]. It is also known that mortality is increased after suffering hip fracture. Furthermore, mortality of nursing home residents with hip fracture compared to residents without hip fracture is markedly higher, too [18].

The aim of this study was on the one hand to estimate incidence rates (IR) of hip fractures in nursing homes residents stratified by sex, age, level of care and the time period after nursing home admission. On the other hand, we aimed to evaluate mortality of residents with hip fracture compared to residents without.

## 2. Materials and Methods

### 2.1. Data Source and Study Population

For this study, data of a major German statutory health insurance fund (DAK-Gesundheit Hamburg, Germany) were used. In 2017, 71.1 million persons are insured in the statutory health insurance in Germany and the DAK-Gesundheit is one of Germany’s largest funds with about 5.8 million members [19]. The dataset consisted of all people aged ≥65 years newly admitted to a nursing home between 1 January 2010 and 31 December 2014. This means that 365 days before admission to a nursing home no benefits for inpatient care were billed to the persons and they had to be insured throughout this time with the DAK-Gesundheit.

We used data on sex, year of birth, date of admission or exit to a nursing home and date of possible death. In addition, data on duration of the insurance period as well as information on possible hospital stays were available. These data included date of admission and discharge and discharge diagnoses based on the 10th revision of the International Classification of Diseases (ICD-10). In 1995, a long-term care insurance was introduced in Germany, which is compulsory for all citizens. The classification of persons needing care is done by the medical service of the German statuary health insurance system (MDK). Information about claiming of benefits in certain time periods that could be assigned to a certain level of care were available in a further dataset. Thus, information on the level of care at nursing home admission and possible change in level of care could be determined. People that require daily help for 1.5, 3 or 5 h are categorized in level of care ‘I’, ’II’ or ‘III’. Corresponding to the level of care daily help must be required in activities like washing, eating or dressing for at least 45 min (level ‘I’), 2 h (level ‘II’) or 4 h (level ‘III’). In the dataset 1088 residents were categorized in level of care ‘0’ identifying persons with significantly reduced skills in daily life and high need of supervision. Because of the comparable need of care, level of care ‘0’ and ‘I’ were combined in this sample. All datasets used could be linked by an identification number and thus be clearly assigned to an insured person. The data were anonymous and we performed a complete case analysis.

### 2.2. Hip Fractures

Our main outcome was hip fracture. Main hospital discharge diagnoses were used to identify hip fractures (ICD-10: S72). The data also showed whether a second or third hip fracture diagnosis of the same person took place during the observation period. To exclude readmissions due to the same fracture, only the first incident fracture during institutionalization was used. This approach was also applied by previous studies [7,8].

### 2.3. Selection of Controls

To compare mortality to an unaffected population, one resident without a hip fracture (unexposed) was matched to each person with a hip fracture (exposed) by sex, age at nursing home admission and level of care using risk-set sampling. Thus, hip fracture cases could also be a control before their hip fracture diagnosis. From this risk set, a random sample was drawn, so that for each case exactly one control was selected (1:1). Survival time for cases started at admission date to hospital because of hip fracture, for controls at index-date (same time period from admission to nursing home until admission to hospital like the corresponding case).

### 2.4. Statistical Analyses

Person-years (PY) at risk were accumulated between date of admission to the nursing home and date of admission to hospital with first hip fracture, end of insurance period, the end of observation period (31 December 2014) or death, whichever came first. The overall IR was calculated by dividing the total number of hip fractures by the total number of PY at risk. IR were stratified by sex, age (5-year-intervals), level of care (at nursing home admission) and time after admission with 95% confidence intervals (95% CI) according to the substitution method [20,21]. Time after admission stratified IR were calculated for each month in the first year after admission by dividing the number of hip fractures that happened in the according month by the cumulated number of PY for this time period. To study differences of hip fractures between males and females according to age and level of care, incidence rate ratios (IRR) with 95% CI were estimated using the Byar method. 

Furthermore, survival in cases with hip fractures and controls was analyzed using the Kaplan-Meier method with 95% CI of Hall-Wellner. Differences between groups were evaluated using the log-rank test. A Cox proportional hazard model was applied to determine factors associated with mortality. Hazard ratio (HR) with 95% CI were estimated. However, the proportional hazard assumption was not met which was tested as an interaction of time and hip fracture as a time-dependent covariable in the Cox model [22]. We then performed Cox Regression using discrete time intervals to model the time dependency of hip fracture and to evaluate predictors for death in multivariate analyses. As predictors we included the following independent variables: group (cases and controls), interaction of hip fracture with the discrete time intervals (1–2, 3–4, 5–6, 7–12 and >12 months), sex (female and male), age (65–74, 75–84, 85–94, 95+ years) and level of care (0/I, II, III). 

Non-overlapping 95% CI or *p*-values ≤ 0.05 were considered statistically significant. OpenEpi version 3.01 (Open Source Epidemiologic Statistics for Public Health, Atlanta, GA, USA) was used for estimating IRR, all other analyses were carried out with SAS version 9.4 for Windows (SAS Institute Inc., Cary, NC, USA). 

## 3. Results

### 3.1. Baseline Characteristics

Overall 127,227 persons were included. The cohort was made up of 94,904 women (74.6%) and 32,323 men (25.4%). The mean age was 84.0 years (84.5 and 82.5 years in women and men). Female residents were categorized into levels of care ‘I’, ‘II’ and ‘III’ in 60.7%, 32.9% and 6.3% and male residents in 50.0%, 40.6% and 9.5%, respectively.

### 3.2. Incidence of Hip Fracture

During an observation time of 168,588 PY a total of 8537 residents had at least one hip fracture, which results in an overall IR of 50.6/1000 PY. The IR in women was significantly higher than in men (52.9 vs. 42.5/1000 PY) (Table 1). 

Generally, IR increased with raising age. Just in women IR declined in the highest age category (≥95 years). At the age of 65–69 years women’s risk to gain a hip fracture was 1.42 higher compared to men but the difference decreased with rising age and disappeared in those aged 90 years and older (Table 1). 

Low levels of care at nursing home admission were associated with a higher risk of hip fracture, both in women and men. While IR in level of care ‘0/I’ and ‘II’ were almost similar, IR in level of care ‘III’ decreased strongly. Furthermore, women had a higher risk of hip fracture in all categories of level of care compared to men and the IRR remained nearly unchanged (1.22 to 1.27) (Table 1).

**Figure 1:**
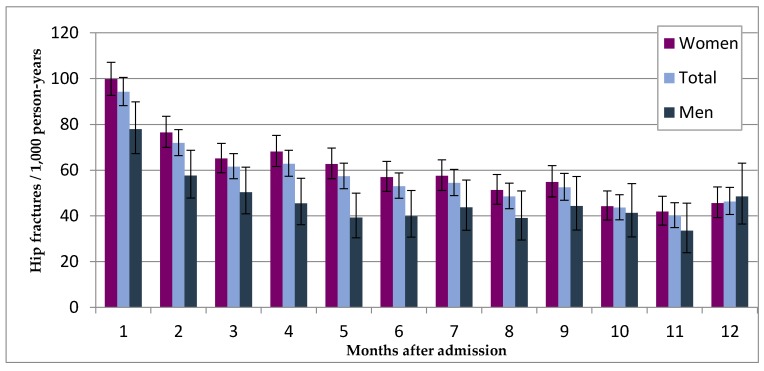
Incidence rates of hip fractures stratified by the period after nursing home admission with 95% CI, by sex.

The IR was highest in the first month after nursing home admission (94.2/1000 PY) and was almost twice the overall IR, both for women and men compared to the whole observation period (50.6/1000 PY). After a five months period of staying in a nursing home the IR reached a plateau that remained nearly unchanged at the level of the overall IR (Figure 1 and Table A1). Overall, 23.5% of female’s, 26.7% of male’s and 24.1% of all hip fractures took place in the first three months after admission. After nine months 50.2% of all 8537 observed hip fractures had happened.

### 3.3. Mortality after Hip Fracture Compared to Controls

For two residents with hip fracture no suitable control could be found. Thus, 8535 case-control pairs remained for analyses. 82.0% of cases were women and mean age was 85.3 years. Because of matching, sex, age and level of care were equally distributed in cases and controls.

**Figure 2:**
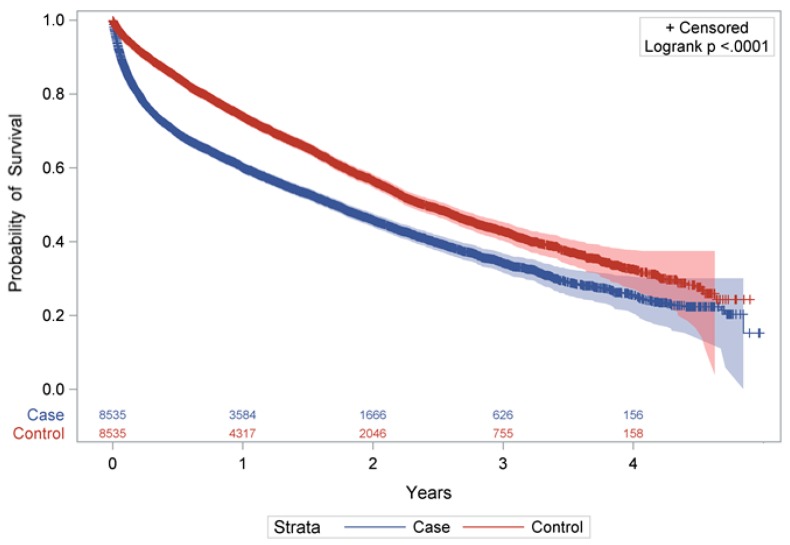
Kaplan–Meier-Plot: Survival probability of the hip fracture group (*n* = 8535) and in controls (*n* = 8535) with 95% Hall–Wellner Bands and number of individuals at risk.

As shown in Figure 2, persons with a hip fracture had a significantly lower probability of survival than residents without (*p* < 0.0001). There were also considerable differences in the median survival time between cases and controls (1.7 to 2.4 years) (Q1: 0.3 vs. 0.9 years and Q3: 4.0 vs. 4.6 years). For cases, one year after the fracture mortality was 39.9%, which was 1.53-fold increased compared to controls with 26.1% (Table 2) (after 4 years: 74.6% vs. 67.4%). The difference between the groups was particularly noticeable in the first six months after hip fracture and was found in both sexes. 

Mortality seemed to be different at different time periods after the injury. The proportional hazard assumption was violated: there was a significant time dependency of hip fracture (*p* < 0.0001) and this was also found in both sexes. The relative mortality risk after hip fracture, adjusted for sex, age and level of care is shown in Table 3. In the first two months after index-date mortality was increased about nearly three times (HR: 2.82; 95% CI 2.57–3.11). HRs declined afterwards and after six months there was no difference in mortality between cases and controls anymore (HR: 1.10; 95% CI 0.98–1.22). In addition, sex, age and level of care were also associated with mortality. There was no notable difference between women and men.

## 4. Discussion

The findings of this study provide important information on hip fracture epidemiology of nursing home residents and confirm previous studies with high IR for this population [4,8,9,15,23,24,25,26,27]. Compared to Icks et al. [13] estimation of hip fractures in Germany’s total population the overall IR of people ≥65 in the present study was increased 7.6 times. Although Icks et al. estimated cumulative incidences and included only proximal femur fractures (ICD-10 S72.0 to ICD-10 S72.2), the large difference in IR cannot be solely explained by this. Defining hip fractures the same way in the present study, 8.1% of hip fractures would have been excluded. In addition, a higher mean age of the nursing home population is likely and has to be considered. However, fracture rates of the nursing home population are included in the fracture rates of the German total population. Thus, the real difference is likely to be higher. Higher IR compared to other studies of nursing home populations like e.g., Rapp et al. [7] could be explained by including only newly admitted residents in our study.

We observed a higher risk for female nursing home residents compared to males confirming numerous previous studies [7,8,27,28,29]. The IRR between women and men decreased with rising age consistent to previous studies [7,8]. Previous analyses showing no differences between sexes or even higher rates for men could be confounded by the functional status of an individual. A high degree of mobility is associated with falls and hip fractures compared to bedridden patients. For example, in Berry’s et al. [9] study population, men showed a better functional status than women (in contrary to the present study). Assuming that hip fracture rates increase with rising functional status, men in the study by Berry et al. and women in the present study had a higher risk. This could explain to some extent the differences between the two studies. Furthermore, women’s average age was two years higher which further increased the risk of hip fracture. 

However, risk of hip fracture in residents with level of care ‘I’ and ‘II’ was almost similar, both in women and men confirming findings of Rapp et al. [15]. A clearly lower risk in persons with level ‘III’ was noticeable, too [8,9,14,15]. Residents categorized at level of care ‘I’ at admission had a nearly two times higher IR than residents at level ‘III’. However, a nearly linear association between risk of hip fracture and level of care, like observed by Rapp et al. [8], could not be confirmed. Differences in IR in all levels of care are pronounced in all age categories and in both sexes (data not shown). These findings might indicate that a high proportion of residents with level of care ‘III’ seem to be bedridden and thus have a low risk of fracturing a hip compared to residents with level of care ’I’ or ‘II’, who are mobile.

We also observed increasing IR with rising age which confirmed previous studies [10,11,12,13], regardless of sex. Just in the highest age category ≥95 years a slight decrease was observed in women. This effect was also found by Rapp et al. [7]. Furthermore, a stronger increase in rates at the age of 70 to 74 years in women and from 75 to 79 years in men was found. 

Just a few studies have analyzed the time period between nursing home admission and the occurrence of hip fractures. In Germany, two studies have shown that IR in the first months after admission to a nursing home are highest [8,15]. We confirmed these results in the present study. Overall, IR in the first month after admission was 94.2/1000 PY and subsequently declined afterwards until it reached the level of the overall IR after five months. Compared to men, IR of women in the first month after admission is higher but after ten months IRs of women and men were almost similar. Nevertheless, about 25% of all hip fractures took place in the first three months after admission and about 50% after nine months. These facts could be explained by the challenge of residents’ new living environment after admission. Only when residents get used for example to their new room, the way to the bathroom or other new aspects of their living environment their risk in falling reduces. This adaptation to the new living conditions is harder with certain comorbidities like dementia, which is highly prevalent in residents of nursing homes [30,31]. However, another possible explanation seems to be the generally poorer residents’ condition of health at the time of admission. The majority of these people was released previously from hospital or may have functionally deteriorated at home so that an outpatient nursing service was no longer sufficient. Thus, high IR in the first few months after admission seem as plausible as the decline in IR with continuing stay in a nursing home. It is to be assumed that a person has a lower risk if he or she has spent a certain time in a nursing home without a hip fracture.

We observed significant differences in mortality between residents with hip fracture and residents without, both for women and men. To assess the time dependency of these differences we analyzed the interaction of hip fracture with discrete time intervals after index-date. Two months after hip fracture mortality of cases was almost three times higher than of their matched controls. Mortality in cases subsequently declined and after six months there was no difference in mortality between persons with and without hip fracture anymore. A similar pattern in a nursing home population was observed by Rapp et al. [8] where mortality in women was increased in the first three months after index-date (men: in the first six months). However, although most other studies reported a stronger effect of the hip fracture on mortality in the first six months this effect persisted for several years thereafter [17,18,32,33,34,35,36] We also showed a higher mortality for men than for women. Expectedly, risk of dying increased with rising age and increasing level of care.

In the present study all inpatient hip fractures (main hospital discharge diagnosis) of a large German health insurance company were recorded. Thus, a major strength is the large number of participants making it possible to calculate stratified analyses precisely. Furthermore, the exact time periods for being under risk of hip fracture could be calculated.

Some limitations have to be considered mainly relying on the data used. Several factors may contribute to the increase in mortality after hip fracture including postoperative events associated with hip surgery like cardiovascular or pulmonary complications or infections [37,38,39]. We do not know if and to which extent the higher mortality rates are driven by these complications. In addition, clinical measures like functional and cognitive status were not available due to the nature of the data.

We had no information if a person had a hip fracture before the admission to a nursing home. Thus, it could be possible that the first hip fracture in nursing home was a readmission of an incident fracture before the admission to nursing home. And because individuals who had already a hip fracture have a higher risk of gaining another hip fracture [40] the IR could be overestimated. But including only the first fracture of a resident in a nursing home and also only main hospital discharge diagnosis could have led to an underestimation of the IR.

The representativeness of the data also needs to be discussed, since the statutory health insurance covers only about 90% of the German population. Ten percent of the population is insured with private health insurance and could not be included in the analysis. Furthermore, it cannot be estimated whether and to what extent the nursing home cohort of the insured population of the DAK-Gesundheit deviates from the general German nursing home population. The higher IR of the present study could indicate structural differences of the DAK-Gesundheit compared to other statutory health insurance funds and thus lead to an overestimation of the risk [41]. 

## 5. Conclusions

The study found that risk of hip fractures in nursing home residents is high. IR increased with rising age and decreased with increasing level of care. IR in women were higher than in men but the IRR decreased with rising age. IR were highest in the first months after nursing home admission and subsequently declined afterwards. Mortality in residents with hip fracture was significantly higher compared to residents without hip fracture. 

The findings of this study can help to gain a better understanding of the patterns of hip fracture development in nursing homes and thus to develop appropriate preventive approaches. Awareness should be raised immediately after admission to the nursing home because fracture rates are highest in the first months.

Further research should examine comorbidities and patterns that are involved in experiencing a hip fracture, especially in the setting nursing home. Furthermore, future research on the epidemiology of hip fractures in nursing home residents with hip fracture should always include analyses stratified by sex, age and time period between nursing home admission and the occurrence of hip fracture. 

## Figures and Tables

**Table 1:** Age- and level of care stratified hip fracture rates * in newly admitted nursing homes residents between 2010 and 2014, by sex.

Men				Women			IRR Women/Men (95% CI)
	*N* ^†^	PY	IR ^☐^ (95% CI)	*N* ^†^	PY	IR ^☐^ (95% CI)	
**Age (years)**							
65–69	54	2440	22.1 (16.6–28.9)	128	4074	31.4 (26.2–37.4)	1.42 (1.04–1.96)
70–74	116	4627	25.1 (20.7–30.1)	332	10,158	32.7 (29.3–36.4)	1.30 (1.06–1.62)
75–79	188	6222	30.2 (26.0–34.9)	698	16,949	41.2 (38.2–44.4)	1.36 (1.16–1.60)
80–84	364	8568	42.5 (38.2–47.1)	1448	28,733	50.4 (47.8–53.1)	1.19 (1.06–1.33)
85–89	470	8887	52.9 (48.2–57.9)	2497	42,418	58.9 (56.6–61.2)	1.11 (1.01–1.23)
90–94	282	4493	62.8 (55.7–70.5)	1555	24,518	63.4 (60.3–66.7)	1.01 (0.89–1.15)
≥95	64	962	66.6 (51.3–85.0)	341	5540	61.5 (55.2–68.4)	0.92 (0.71–1.21)
**Level of care**							
0/I	948	21,060	45.0 (42.2–48.0)	4937	89,880	54.9 (53.4–56.5)	1.22 (1.14–1.31)
II	538	12,852	41.9 (38.4–45.6)	1909	37,214	51.3 (49.0–53.7)	1.22 (1.11–1.35)
III	52	2286	22.7 (17.0–29.8)	153	5296	28.9 (24.5–33.8)	1.27 (0.93–1.75)
**Total**	1538	36,198	42.5 (40.4–44.7)	6999	132,390	52.9 (51.6–54.1)	1.24 (1.18–1.32)

* First hip fracture after nursing home admission. ^†^ Number of hip fractures. ^☐^ per 1000 person-years. PY, person-years; IR, incidence rate.

**Table 2:** Mortality in the hip fracture group (*n* = 8535) and in controls * (*n* = 8535) with 95% CI in different periods after index-date.

	Women		Men		Total	
	Cases	Controls	Cases	Controls	Cases	Controls
Mortality in % (95% CI)						
0.5 year	27.6	14.3	44.3	21.3	30.6	15.6
	(26.6–28.7)	(13.5–15.2)	(41.7–46.8)	(19.9–23.6)	(29.6–31.7)	(14.8–16.4)
1 year	36.7	24.1	54.3	35.4	39.9	26.1
	(35.5–38.0)	(23.0–25.2)	(51.7–57.0)	(32.9–38.1)	(38.8–41.1)	(25.1–27.1)
2 years	51.2	40.6	67.4	57.1	54.1	43.5
	(49.8–52.6)	(39.2–42.0)	(64.6–70.2)	(54.0–60.3)	(52.8–55.4)	(42.2–44.8)
3 years	63.1	54.4	78.1	70.8	65.8	57.2
	(61.5–64.8)	(52.6–56.1)	(74.9–81.2)	(67.2–74.4)	(64.3–67.3)	(55.6–58.8)
4 years	71.7	65.1	88.2	78.3	74.6	67.4
	(69.6–73.9)	(62.7–67.7)	(83.7–91.9)	(73.8–82.6)	(72.7–76.5)	(65.3–69.6)

* Matched for sex, age, level of care and time under risk.

**Table 3:** Multivariable adjusted mortality risk in the hip fracture group (*n* = 8535) and in controls * (*n* = 8535) with 95% CI.

	Women (HR (95% CI))	Men (HR (95% CI))	Combined (HR (95% CI))
**Cases vs. Controls**			
x 1–2 months	2.65 (2.37–2.99)	3.34 (2.78–4.01)	2.82 (2.57–3.11)
x 3–4 months	1.92 (1.65–2.23)	2.07 (1.58–2.72)	1.96 (1.72–2.23)
x 5–6 months	1.48 (1.24–1.76)	1.35 (0.96–1.92)	1.46 (1.25–1.70)
x 7–12 months	1.11 (0.98–1.26)	1.05 (0.83–1.33)	1.10 (0.98–1.22)
x >12 months	1.05 (0.96–1.14)	0.94 (0.78–1.13)	1.03 (0.95–1.11)
**Sex (men vs. women)**	-	-	1.72 (1.62–1.81)
**Age**			
65–74 years	1	1	1
75–84 years	1.34 (1.17–1.53)	1.50 (1.24–1.82)	1.40 (1.25–1.56)
85–94 years	1.89 (1.66–2.15)	2.06 (1.71–2.47)	1.95 (1.76–2.17)
≥95 years	2.70 (2.30–3.16)	3.08 (2.36–4.04)	2.82 (2.47–3.23)
**Level of care**			
0/I	1	1	1
II	1.23 (1.17–1.30)	1.22 (1.11–1.35)	1.12 (1.17–1.29)
III	1.74 (1.50–2.03)	1.31 (1.02–1.69)	1.60 (1.40–1.82)

* Matched for sex, age, level of care and time under risk.

## Appendix A

**Table A1:** Time after admission stratified hip fracture rates * in newly admitted nursing homes residents between 2010 and 2014, by sex.

Women					Men			Total		
	*N* ^†^		PY	IR ^☐^ (95% CI)	*N* ^†^	PY	IR ^☐^ (95% CI)	*N* ^†^	PY	IR ^☐^ (95% CI)
Months after admission										
1		732	7345	99.7 (92.6–107.1)	190	2439	77.9 (67.2–89.8)	922	9784	94.2 (88.2–100.5)
2		509	6652	76.5 (70.0–83.5)	123	2137	57.6 (47.8–68.7)	632	8789	71.9 (66.4–77.7)
3		403	6194	65.1 (58.9–71.7)	98	1947	50.3 (40.9–61.3)	501	8140	61.5 (56.3–67.2)
4		398	5842	68.1 (61.6–75.2)	82	1803	45.5 (36.2–56.5)	480	7645	62.8 (57.3–68.7)
5		348	5546	62.7 (56.3–69.7)	66	1681	39.3 (30.4–50.0)	414	7227	57.3 (51.9–63.1)
6		301	5286	56.9 (50.7–63.8)	63	1578	39.9 (30.7–51.1)	364	6864	53.0 (47.7–58.8)
7		291	5059	57.5 (51.1–64.5)	65	1488	43.7 (33.7–55.7)	356	6546	54.4 (48.9–60.3)
8		248	4837	51.3 (45.1–58.1)	55	1406	39.1 (29.5–50.9)	303	6244	48.5 (43.2–54.3)
9		254	4632	54.8 (48.3–62.0)	59	1331	44.3 (33.8–57.2)	313	5963	52.5 (46.8–58.6)
10		196	4433	44.2 (38.2–50.9)	52	1260	41.3 (30.8–54.1)	248	5693	43.6 (38.3–49.3)
11		178	4245	41.9 (36.0–48.6)	40	1195	33.5 (23.9–45.6)	218	5440	40.1 (34.9–45.8)
12		185	4054	45.6 (39.3–52.7)	55	1134	48.5 (36.5–63.1)	240	5189	46.3 (40.6–52.5)

* First hip fracture after nursing home admission. ^†^ Number of hip fractures. ^☐^ per 1000 person-years. PY, person-years.
